# Editorial: Evolving strategies in Lyme borreliosis treatment and prevention

**DOI:** 10.3389/fmed.2026.1926686

**Published:** 2026-07-24

**Authors:** Renata Šmit, Ying Zhang

**Affiliations:** 1 University Medical Center Groningen (UMCG), Groningen, Netherlands; 2 Institute for Persistent Infections and Antibiotic Resistance, Dongguan Innovation Institute, School of Medical Technology, Guangdong Medical University, Dongguan, China

**Keywords:** diagnosis, epidemiology, integrated modeling, Lyme borreliosis, patient-centered research, persistent symptoms, Post-Treatment Lyme Disease Syndrome (PTLDS), tick-borne diseases

Tick-borne diseases continue to challenge clinicians, researchers, and public health systems. This Research Topic brings together six articles that examine these challenges from different but complementary perspectives: classification, clinical follow-up, treatment of persistent symptoms, epidemiology, vaccine modeling, and the patient and economic burden of chronic tick-borne illness. Although Lyme borreliosis is the central focus, the contributions also highlight broader issues shared across many tick-borne infections, including diagnostic limitations, persistent symptoms, and the need for improved prevention and treatment strategies.

Together, these studies provide a comprehensive and timely overview of the current landscape in Lyme borreliosis research—demonstrating how improved classification systems, long-term clinical follow-up, careful evaluation of emerging treatments, expanded epidemiological insight, advanced modeling approaches, and real-world patient-centered analyses collectively strengthen our ability to understand, prevent, and manage both acute and persistent manifestations of tick-borne diseases.

The first article, by Fallon et al., presents a research-focused classification system for Lyme disease. The authors note that many studies have used narrow inclusion criteria that do not reflect the range of patients seen in practice. Their system distinguishes early Lyme disease, objective complications such as arthritis or neurological involvement, and persistent symptom syndromes such as Post-Treatment Lyme Disease Syndrome (PTLDS). This classification system supports clearer reporting and improves comparability across studies, especially those examining infection-associated chronic illness.

The second article, from the Lyme Disease Biobank, reports 10 year follow-up data from individuals with early Lyme disease (Horn et al.). The study confirms that standard two-tier serology is often negative in early infection and that IgG seroconversion is uncommon after treatment. Although most participants improved, about one in five—or 20%—continued to report symptoms such as fatigue, joint pain, and muscle pain 3 months after therapy. These findings highlight the need for better diagnostic tools, routine follow-up, and improved treatment, as persistent symptoms are not rare even after timely antibiotic treatment.

The third article describes a small pilot trial of disulfiram for nine individuals with persistent symptoms after treated Lyme disease (Kuvaldina et al.). Although some participants experienced meaningful improvement in fatigue and quality of life, the medication also caused notable or even severe side effects, including liver abnormalities. Only three of nine participants completed the full 8-week treatment course. Efficacy could not be assessed due to the small sample size and lack of a placebo-control group. The study was terminated early partly due to higher-than-expected adverse events. This study illustrates both the potential and the limitations of disulfiram and underscores the importance of evidence-based evaluation of treatments for persistent symptoms with careful attention to safety.

The fourth article reviews bacterial tick-borne febrile illnesses in Africa (Adamu et al.). The authors document the presence of pathogens such as *Anaplasma, Ehrlichia, Bartonella, Borrelia, Coxiella burnetii*, and *Francisella* across multiple countries. Many infections are under-recognized because symptoms overlap and diagnostic tools are limited. Misdiagnosis is common, particularly in regions where malaria and other febrile illnesses are prevalent. The review highlights the need for improved diagnostic capacity and more epidemiological research to clarify the burden of tick-borne diseases on the continent.

The fifth article introduces a novel integrated Markov-based model for evaluating the cost-effectiveness of a combined Lyme borreliosis and tick-borne encephalitis vaccine (Šmit). Building on her earlier work developing the first cost-effectiveness model for tick-borne encephalitis vaccination (1), Šmit now extends this foundation into a fully integrated dual-pathogen framework that jointly evaluates Lyme borreliosis and tick-borne encephalitis within a single interacting structure. This new generation model incorporates clinically aligned disease states, realistic transition pathways, and co-infection dynamics, offering a more accurate representation of the complex natural history of tick-borne diseases. By unifying dual-pathogen mechanics with cross-regional epidemiological assumptions, this modeling approach restores the internal mathematical validity, provides a robust foundation for coordinated prevention planning, and underscores the growing need for integrated global strategies that align surveillance, diagnostics, and vaccine policy across borders. The structure of this framework is adaptable and could be expanded not only to additional tick-borne pathogens but also to other infectious diseases with similar natural history and vaccination dynamics.

The sixth article expands the Research Topic by presenting the first Irish patient-centered analysis of chronic tick-borne illness and Post-Treatment Lyme Disease Syndrome (PTLDS). Avramovic et al. combine patient-reported outcomes, healthcare-utilization data, disease modeling, and a novel patient-journey roadmap to quantify the economic burden and care fragmentation experienced by patients with persistent symptoms. Their findings reveal long diagnostic delays, extensive “doctor-to-doctor wandering,” significant out-of-pocket costs, and substantial impacts on employment. The study also proposes two methods for estimating PTLDS prevalence in Ireland, highlighting major gaps in national surveillance and the urgent need for improved data collection, clinical pathways, and public health planning.

Together, these six articles highlight several shared themes. Accurate classification is essential for research, diagnosis, and modeling. Early Lyme disease remains difficult to diagnose with current tests, and structured follow-up is important because a meaningful proportion of patients continue to experience symptoms. Persistent symptoms after Lyme disease are clinically relevant and require safe and effective treatment options. Diagnostic gaps affect not only Lyme disease but many other tick-borne infections worldwide. Real-world patient-journey analyses underscore the systemic challenges faced by individuals with chronic symptoms. Finally, modeling frameworks must reflect clinical reality if they are to guide vaccine development and public health decisions.

This Research Topic demonstrates progress across these areas. By improving classification, diagnostics, clinical understanding, patient-centered research, and modeling, these contributions strengthen the foundation for future research and support the development of more effective and integrated strategies to prevent and manage Lyme borreliosis and other tick-borne diseases. Taken together, these findings underscore the urgent need for coordinated, integrated global strategies that align surveillance, diagnostics, clinical care, and prevention efforts across regions and health systems to address the growing burden of tick-borne diseases worldwide.
